# Australian radiation therapy – Part two: Reflections of the past, the present, the future

**DOI:** 10.1002/jmrs.40

**Published:** 2014-01-23

**Authors:** Susan Merchant, Grad Cert Grief & Pall Care Counselling, MIR, Georgia Halkett, Charlotte Sale

**Affiliations:** 1 Radiation Oncology Department, Royal Adelaide HospitalAdelaide, SA, Australia; 2 Curtin Health Innovative Research Institute, Curtin UniversityPerth, WA, Australia; 3 Radiation Oncology, Andrew Love Cancer Centre, Barwon HealthGeelong, Vic., Australia

**Keywords:** Australian history, radiation therapists, radiotherapy

## Abstract

**Introduction:** Documentation on the history of Australian radiotherapy is limited. This study provides radiation therapists' (RTs) perspectives of the people, workplace, and work practices in Australian radiotherapy centres from 1960 onwards. It provides a follow-up to our previous study: *Australian radiation therapy: An overview – Part one*, which outlines the history and development of radiotherapy from conception until present day.

**Methods:** Four focus groups were conducted on separate occasions in 2010, one in South Australia and three in Victoria, Australia. Participants who worked in radiotherapy were purposively selected to ensure a range of experience, age, and years of work.

**Results:** From a RT perspective, radiotherapy has evolved from a physically demanding ‘hands-on’ work environment, often with unpleasant sights and smells of disease, to a more technology-driven workplace.

**Conclusion:** Understanding these changes and their subsequent effects on the role of Australian RTs will assist future directions in advanced role development.

## Introduction

Australian radiotherapy is now recognised as an essential treatment modality with more than 52% of all cancer patients recommended for radiotherapy.^1^

Changes have occurred in disease presentation, general attitudes towards cancer, new technology, radiation safety, communication, information, and education. Understanding the significance of the historical development of radiotherapy on the roles of radiation therapists (RTs) is important for future developments in the profession. It will provide insight and direction for education, communication, patient supportive care, and continuing professional development (CPD).

Our previous study (*Part one*) described the chronological development of radiotherapy.^2^ This subsequent study explores the perspectives of Australian RTs on the different facets of radiotherapy, the role of the RT, and the impact changes have made on the profession. The background provides the major historical events highlighted in *Part one* to emphasise the significance of these changes on the development of radiotherapy and the RT role.

## Background

The first hundred years of radiotherapy saw many radical changes in equipment and treatment techniques. Expectations of the personnel performing the treatments also changed. Medical radiations became a new paradigm of health care requiring trained workers to operate the equipment.

### Prior to 1960

Radiotherapy in Australia began soon after the discovery of radiation was announced. Attempts in the early 1900s were made by dermatologists to treat skin tumours.^3^,^4^ Around the beginning of the second decade orthovoltage (deep X-ray) was introduced providing greater treatment scope. The installation of machines and the number of operators increased throughout Australia creating a need for the development of radiation monitoring standards in Australia.^5^

Post–World War Two, the increased knowledge of radiation led to the development of new equipment and treatment delivery with Cobalt-60 machines and linear accelerators becoming the modern treatment modalities for radiotherapy.^5^,^6^

### 1960–2010

By 1960 many radiotherapy departments were staffed by a combination of radiation oncologists (ROs), RTs, nurses, physicists, and engineers. RTs were trained specifically in radiotherapy unlike in previous decades.

The introduction of computed tomography (CT) and magnetic resonance imaging (MRI) paved the way for more accurate and complex treatment planning and delivery.^7^–^10^ Electronic management of data coupled with record and verify systems have ensured increased safety standards for both patients and treatment providers. The introduction of dynamic wedges and multileaf collimators (MLCs) provided greater accuracy in treatment delivery and decreased the physical demands on RTs by reducing the use of both hard wedges and shielding blocks.

‘Cancer’ was often equated with a death sentence. Previously the oncologist would refer to a patient's disease as a lump or tumour to dispel any fears the patient had about their health.^7^ However, improved treatment outcomes continue to change the general public's perception of cancer. National screening and awareness campaigns continue to promote health checks for cancer and precancerous conditions.

Education of RTs has evolved from on-the-job training to a university degree with postgraduate opportunities. The emergence of university graduates has increased awareness and involvement in research activities within the profession. Australian RTs are publishing articles in internationally renowned journals, a further indication of the educational changes within the profession.

The changes in approaches to planning and treatment delivery have impacted on the people, workplace, and work practices that comprise the radiotherapy profession. It is on this basis the authors sought RTs' perspectives of the changes in radiotherapy from 1960 onwards to gain a greater understanding of these changes and the resulting impact on the RT role.

## Methods

A qualitative research approach consisting of four focus groups was used to conduct this study. This study contributed to a larger ethnographic study conducted by SM on RTs' interactions with their patients. Data analysis was informed by ethnographic principles and the conceptual frameworks are described later in this article.

Ethics approval was gained from Curtin University HREC (HR RD 13.10) prior to recruitment and written consent was attained from all participants prior to the start of each focus group.

### Conceptual framework

The researchers have adopted the approach taken by Hacking,^11^ who draws on both Goffman and Foucault, to understand people from both a sociological and an archaeological perspective respectively. The approach of Hacking is suitable because the ethnographic method is concerned with behaviour and actions of a group of people in order to understand the group's culture.^11^ Hacking suggests that these two theorists' concepts put forward about people, their actions and the space where the actions occur are not opposite, but rather they complement each other. These concepts provide a vehicle for understanding how the past culture and perspectives of a group informs the present and the future perspectives and undertakings of a group of people. Interactions between members of the group and others outside of that group also impact on the group's perspectives and changes in behaviour, actions, and the space where these take place. Therefore, concepts of Foucault and Goffman were adopted because both of these theorists are concerned with institutions and the work and interactions of a group of people that takes place within institutions; Foucault on a macrosocial level and Goffman on a microsocial level and were deemed appropriate because the study centres on RTs and their culture, work, and interactions that take place within the radiotherapy environment.^11^–^14^

### Study design

Focus groups were an appropriate exploratory method to gather perspectives about radiotherapy shared by RTs. Focus group interviews allowed exploration of different facets of radiotherapy and the impact of historical changes on radiotherapy and RTs.^15^–^17^ Focus groups were held on four separate occasions in 2010, one in South Australia and three in Victoria. The groups were asked general questions about their current and past experiences and memories of daily work in radiotherapy departments during their working life.

At the time of data collection all three authors were employed by separate institutions: S. M. was employed as a clinical treatment RT; G. H. was a senior research fellow (cancer and radiotherapy); C. S. (RT) was involved in both clinical and educational roles in a radiotherapy centre.

### Participants

Participants were purposively selected to ensure a range of age, experience, and years of work in radiotherapy. Two focus groups consisted of RTs with clinical radiotherapy-related work experience ranging from 12 years to more than 40 years and two focus groups consisted of RTs with 5 years or less experience (see Table 1 for more detail). Each focus group met on a single occasion and was digitally audio-recorded by SM and hand-written notes were taken by G. H. or C. S. to supplement the data collection and assist in the data analysis.

**Table 1:** Demographics of focus group participants.

Year commenced work in radiotherapy	Currently employed in clinical radiotherapy (April–June 2010)^1^	Total number of years worked in radiotherapy	Years worked in Australian radiotherapy	Qualifications
1960	No/retired	47	47	Certificate
1960	Yes	45	43	Certificate
1969	No	39	39	Cert/Ass Dip
1969	Yes	33	33	Cert/Ass Dip
1972	Yes	21	21	Ass Dip
1972	Yes	33	32	Ass Dip/Dip/Masters
1975	Yes	33	33	Ass Dip
1983	Yes	27	17	Degree (UK)^2^
1983	Yes	20	20	Ass Dip
1989	Yes	19	19	Dip
1994	No	12	12	Degree
2005	Yes	5	5	Degree/Grad Cert/Masters
2006	Yes	4.5	4.5	Degree
2006	Yes	4.5	4.5	Degree
2007	Yes	3.5	3.5	Degree
2007	Yes	3.5	3.5	Degree
2008	Yes	2.5	2.5	Degree
2008	Yes	2	2	Masters
2009	Yes	1.5	1.5	Degree
2010	Yes	0.5	0.5	Degree

Ass Dip, Associate Diploma; Cert, Certificate; Dip, Diploma; Grad Cert, Graduate Certificate.

1 Currently clinical in a South Australian or Victorian radiotherapy centre at the time of participation in focus groups.

2 Only participant educated in radiotherapy outside of Australia.

### Data analysis

Data analysis was informed by the ethnographic iterative process. The audio-recordings were transcribed and transcripts were read and re-read to enable themes and subthemes to emerge from the data. Common themes were then extracted from the recordings and transcriptions of the focus groups.^18^,^19^ Each quote is referenced by group number and transcript line number(s), for example, Gr 1 # 10–12.

The concepts of Foucault and Goffman about people, their actions, and the space where the actions occur were used to inform the categories selected and to sort the themes and subthemes that emerged from the data analysis: people, place, and practices.

## Results and Discussion

**Figure 1:**
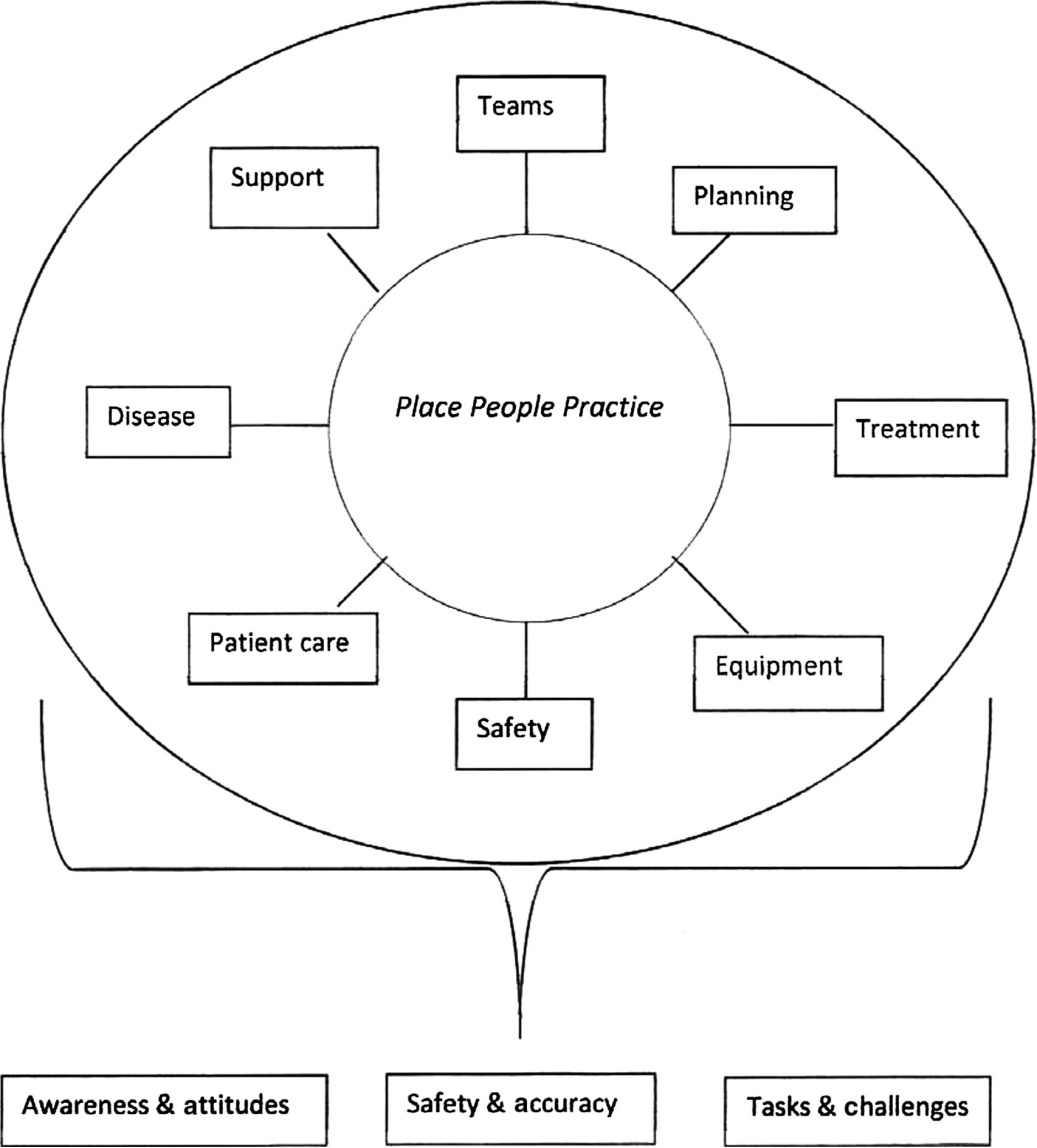
Conceptual framework depicting the eight subcategories and the three predominant themes from data analysis.

Demographic details of participants are provided in Table 1. Eight subcategories of patient care, support, planning, treatment, safety, teams, disease, and equipment emerged from the analysed data. The subcategories were developed and informed by a conceptual framework about people; their actions and the space where the actions occur with three predominant themes of ‘awareness and attitudes’, ‘safety and accuracy’, and ‘challenges’ (see Fig. 1).

### Awareness and attitudes

Awareness of and attitudes towards cancer and radiotherapy treatment combined to form one of three major themes to arise from the findings with further division into three subthemes of ‘cancer as a spoken word’, ‘cancer presentation’, and ‘treatment side effects’.

#### Cancer as a spoken word

Several RTs remarked on the enforced omission of the word ‘cancer’ in the early years of their careers:

*…do you remember when …one could never actually say the word “cancer” to a patient? And then, they say to us, “Have I, have I got cancer?” And we couldn't say yes, no or anything like that*.

Gr1 #1260-1268

*…often the doctor(s) made the decision that they felt psychologically the patient wasn't capable of handling it…they actually made the decision whether the patient was told that they had cancer*.

Gr1 #1271-1288

*We weren't allowed to say “cancer” when we first started…it was a tumour or a lesion*.

Gr2 #482-492

Despite the advances in medical interventions used to treat malignant disease during the mid-1900s, cancer was generally perceived by the public as an incurable disease and the word ‘cancer’ was not openly mentioned by RTs in the presence of patients. RTs were actively discouraged by ROs to say the word cancer.^7^,^20^ Limited communication and information provision to patients was a significant part of the early development of the RT role; reflecting the medical model of health where the focus was placed on the diseased body part of the patient without inclusion of any psychosocial aspects; Foucault referred to this as ‘the medical gaze’.^14^

#### Cancer presentation

Disease presentation and body image were discussed in each focus group. The more experienced RTs spoke of their impressions of the visual and olfaction impact:

*…do you remember the days of the fungating breasts? That smell was…that was so bad. It just looked like cauliflower*.

Gr1 #683

*I can remember maggots in the back, in someone's back*.

Gr2 #2068

*…this man, he came in and he took his glasses off and he had no nose. There was just a nasal septum*.

Gr1 #735

However, the sights and smells of advanced disease, such as fungating breast lesions were encountered less often in current practice according to a Group 3 participant:

*…but people go, oh …back in the day, you got that sort of thing every day …we would have one or two patients on at a time*.

Gr3 #582-584

Two experienced RT participants shared their reflections on notable changes in presentation:

*… the age cohort is very different now. …we would have a few young people and yes we did children, but… most of your patients would probably be considered to a large degree, palliative compared to today*.

Gr2 #2075-2079

*… Presentation of the disease and as a community we've changed our lifestyles…*.

Gr2 #2029

The introduction of focused medical awareness campaigns and continued promotion has assisted the gradual changes in public perception and attitudes towards cancer.^21^ The changes in disease presentation, observed by RTs, are reflective of the changes in the awareness and attitudes to cancer of a more informed community.

#### Treatment side effects

The side effects of radiotherapy treatment were thought to be less extreme than in the past:

…skin reactions… have really decreased …I mean I probably see, like moist desquamation on chest walls…all of that's a bit nasty but, but compared to maybe someone who's been working for, you know, thirty years and they're like, “That's nothing, you know, you should have seen what we used to do to people,”

Gr4 #898-906

*I think the side effects are better these days. … We do IMRT head and neck and their skin is not as red, but they get more dose in their mouth. So they get more sore lips. But the whole effect, they don't get all the mucous*.

Gr1 #4166-4169

The reduction in treatment toxicities is attributed to a combination of earlier disease presentation, improved treatment planning techniques, and advanced interventions.^22^,^23^

### Safety and accuracy

The safe and accurate delivery of radiotherapy is composed of the subthemes of ‘machinery and equipment’, ‘accuracy of treatment delivery’, and ‘radiation safety in the delivery of treatment’.

#### Machinery and equipment

Current delivery of radiation treatment requires strict protocols for the safety of both the patient and the professionals providing the treatment; relying heavily on correctly calibrated machines and accurate planning systems as well as step-by-step checking processes.

Early radiotherapy equipment was described as physically challenging and cumbersome to use:

*Just sort of trying to put a plate on the machine… Yes, the fear of lead*.

Gr 2 #431-433

*The suspended lead with the weight on. Yes, that was hard work. No wonder I've had shoulders fixed*.

Gr2 #437-443

…there was a lever and the patients lying there and you press this lever …we used to come down and touch them. I mean how terrifying. It was enormous wasn't it?

Gr1 #298-302

*…the equipment that never worked … And we all did our backs with it*.

Gr2 #421-423

*But the back pointer you used to have to lift up a bit so you could just get them to match properly*.

Gr1 #573

#### Accuracy of treatment delivery

Previous descriptions provided a basis for comparison with the equipment encountered by the less experienced RTs. A Group 3 participant pointed out that changes occurred very quickly when new technology was introduced into radiotherapy:

*My first year was still looking at films. So within eighteen months, it went from film to no film… I'm right on the crest of when computers took over*.

Gr3 #112-118

A Group 2 participant claimed that the current equipment was not used to capacity:

*Some of the equipment that we use is phenomenal, but a lot of it, we only use a quarter of its potential*.

Gr2 #665

Despite continual changes and development of both equipment and treatment protocols the equipment used by the majority of the RTs was a mixture of both old and new.

#### Radiation safety in delivery of treatment

The radiation safety issues of early practices were raised by Group 1:

*The radioactive plaque (used on) the Superficial: they put this person in the linen cupboard with the plaque on… to keep away from the other patients…they were all going home and somebody said where's this patient? He'd been sitting there all day with this plaque on his hand*.

Gr1 #167-172

Another member of the group raised their concerns about the shielding procedures of vital structures:

*It was a block, mounted, it was actually mounted, it wasn't attached to the machine, it was on a stand and it was sort of put in the way (of the beam)… absolutely archaic when you think about it*.

Gr1 #288-289

The recounts of inaccuracies experienced with equipment use were accompanied by the directives of ROs on how to treat their patients:

I can remember Dr X treating through clothes, saying, “It doesn't matter, just get the dose in there, it doesn't matter.”

Gr1 #908

The safety of specific procedures undertaken in the past was questioned by several participants:

*What about TBI's (Total Body Irradiation)? We had one (patient) that went off the… mechanical thing …the bottom was going too fast and so he lost his balance*.

Gr1 #808

Accurate treatment delivery relies on good quality immobilisation devices. However, immobilisation was a largely underdeveloped area of radiotherapy until the last decade:

*Immobilisation…It just didn't exist did it? (There was) the tape but… if you recall then we actually got quite clever and we used to have the triangles so we could adjust the head to get vertical baselines*.

Gr1 #1151-1154

Group 1 highlighted that the past treatment was simply planned and checked, whereas current treatment requires a number of steps in the checking process:

*…you can't just plan it and have somebody check it. It's got to be checked at this phase… it's got to be checked again at that phase…it's been checked again prior to treatment. So there are more steps in the process*.

Gr1 #3575-3584

### Challenges

The theme challenges consists of two intertwined subthemes: ‘technology challenges’ and ‘the challenges of teamwork’. These subthemes replicate similar findings of Atyeo where the combination of technology and teamwork afford a variety of challenges to team members.^24^

#### Teamwork and technology challenges

The relationships and behaviour of team members, in particular, ROs and senior RTs created challenges for RTs:

*… I can remember the students being terrified and even as a newly qualified, some of them (senior RTs) were horrible*.

Gr2 #1021-1036

*…and often (it) was not just the doctors who were intimidating. There was some senior staff… And …we sort of went in trembling at the knees*.

Gr2 #1134-1141

The recollection of the patriarchal dominance of doctors and senior RTs is reflected in the literature that suggests that the medical model of health endows physicians with power and presents the team as a hierarchical structure.^25^–^27^ Although a hierarchical configuration of the radiotherapy team posed challenges to RTs, further discussion disclosed challenges team members faced when working with each other:

…technology wise, some people really struggle with that, so I think there has to be a massive amount of patience …

Gr4 #560-565

*…I think they're (older RTs) very practical about stuff rather than getting caught up in the technology as well. Getting … like millimetre accuracy and they'll be like, oh, you know, actually you're not going to get that. Yes they look at the bigger picture*.

Gr 4 #576-584

*…you've got a lot to learn from them (older RTs) as well, like they've been in the job a hell of a lot longer than yourself…like the underlying principles of RT … They've got …ideas we probably wouldn't even have thought of*.

Gr4 #569-574

…*they (young RTs) will spend an hour tweaking it for one percent on a patient that's palliative*.

Gr2 #742

*I see eighteen year olds come in and they really have never spoken to an older person. The idea of speaking to an older man with prostate cancer …they wouldn't have a clue what to say*.

Gr1 #2215

Team participation is important in the role of RTs and could impact on the success of team performance. According to Goffman within teams is the existence of a shared reliance between team members with trust and cooperation necessary for successful team performance.^12^

Other concerns were raised by RTs in recent research in 2008.^24^ Atyeo conducted a focus group of RTs, who voiced their unhappiness about the repetitive nature of the work and a lack of challenges and raised concerns that the ‘dynamic’ younger RTs would become bored with the work. The group also expressed concerns that the technology was creating an automated approach to the work. In contrast, Atyeo also reported that RTs with the least experience indicated that they found working with new technology provided satisfaction in the workplace.^24^

#### Technology

Experienced RTs discussed the introduction of new technology:

*I tend to think with the introduction of, new technology, I think Australia actually is wonderful, quickly putting new technology into the clinical setting and making use of it*.

Gr2 #558-561

Technology was discussed as a promoter of change in the roles undertaken by RTs:

*Whereas now with your data image matching… We've become more technical*.

Gr1 #1887-1930

*You ask people to do …IT speciality jobs. …somebody needs to do it and you need to have an IT specialist*.

Gr1 #3531-3534

All these sort of peripherals that…they are part of radiotherapy but you'd almost need a clerical (staff) but you need radiotherapy knowledge …

Gr1 #3545-3548

However, not all technology changes were seen to assist in role development for RTs:

*We introduce new technology into radiotherapy. At no point do we (or) very few times, do we actually sit down and say okay let's take time. We're getting a new machine. What is it able to do? What are we going to be able to improve on our techniques - and how can we change the technique and use everything that's on that new machine to be able to do it. All we do is we adapt our current techniques to fit that machine. Or that machine to fit our current techniques*.

Gr2 #590-598

It was suggested that there are constraints placed on RTs' involvement in research despite ROs striving for improvements and changes to treatment regimes mirroring the findings of Atyeo where a shortage of RTs was used as a reason by some RT managers to stifle further research opportunities of RTs because throughput of patients was the primary concern.^24^

Several RTs voiced concerns about a possible imbalance between patient care and technology in the RT role in contrast to the current drive for a ‘patient-centred’ approach to cancer care:

I still think there's a lot of technology pushed here. …patient-care could be pushed a lot more…

Gr4 #361-365

*I don't think we do a lot of role extension to patient-care. We've certainly pushed a lot of technology development*.

Gr4 #369-372

*…there's a lot of emphasis on the technical side of things rather than of the caring side of things … if you want a career, you want to drive yourself forward, you don't drive yourself forward as a carer, you drive yourself forward as a technician*.

Gr1 #3254-3270

There was concern that less time was given to patient care and more of an expectation of RTs to embrace the technological changes in practice. However, recent research has shown that there is a wide variety in roles undertaken by RTs in Australia and is indicative of a need for structure and standardisation of the practices currently undertaken by RTs.^28^

### Strengths and limitations

The focus groups provided rich data that would be difficult to obtain by other methods of data collection. The first two groups established perspectives from RTs with many years of clinical experience from either private and/or public centres in Victoria and South Australia. A decision was made to conduct further two focus groups to gain the perspectives of recently qualified RTs, once again from either private and/or public centres. However, as a result, this study did not capture the perspectives of RTs that had more than 5 years but less than 12 years of RT experience. The authors acknowledge that further research with a different cohort of participants from a variety of Australian centres in other states could provide other perspectives and add to the credibility of the study.

## Summary

Significant changes in radiotherapy have occurred since the first applications of radiation were used to treat cancer. To gather an understanding of the role of RTs and their culture, work, and interactions that takes place within the radiotherapy environment, Foucault's concepts of institutions, people, and their actions within the institutions (a macrosocial level) and Goffman's concepts of everyday interactions and where the interactions occur (a microsocial level) were used. Focus groups were conducted to elicit the views of RTs on their role and changes in practice from the 1960s to 2010 resulting in three main categories: ‘awareness and attitudes’, ‘safety and accuracy’, and ‘challenges’.

Greater awareness, earlier detection, and better understanding of cancer have contributed to changes in awareness and attitudes of both the general public and health professionals. These changes have been reflected in the development and implementation of radiotherapy practices. The radiotherapy environment, the RTs, and their practices continue to change to ensure the safety and accuracy of treatment delivery and the decrease in side effects.

The demands of work for RTs have changed from a physical ‘hands-on’ environment to a combination of technology and teamwork that provide a cognitively challenging environment. Currently, RTs perceive an imbalance of less patient care in their role with a greater emphasis placed on the development of expertise in the technical and IT aspects of the role.

Exploration of the historical beginnings of the RT role play an important part in determining the future directions of protocols, management, and the expectations of the multidisciplinary team, and the consequent impact on the radiotherapy environment. This study has disclosed an understanding of changes in awareness and attitudes to cancer; the increasing emphasis on safety and accuracy of treatment planning and delivery; and the day-to-day challenges faced by RTs. Understanding the changes that have occurred over time and the impact these changes have had and continue to have on the RT role is paramount for advanced role development in the future.

## Funding Information

S. Merchant was the recipient of the 2008 AIR Research scholarship in Radiation Therapy, a Curtin University Postgraduate Scholarship (CUPS), and Curtin Research Scholarship (CRS). Curtin University also provided additional funding for the focus groups.
